# Maternal MitoQ Treatment Is Protective Against Programmed Alterations in CYP Activity Due to Antenatal Dexamethasone

**DOI:** 10.3390/pharmaceutics17030285

**Published:** 2025-02-22

**Authors:** Millicent G. A. Bennett, Ashley S. Meakin, Kimberley J. Botting-Lawford, Youguo Niu, Sage G. Ford, Michael P. Murphy, Michael D. Wiese, Dino A. Giussani, Janna L. Morrison

**Affiliations:** 1 Early Origins of Adult Health Research Group, Health and Biomedical Innovation, UniSA: Clinical and Health Science, University of South Australia, Adelaide, SA 5000, Australia; millicent.bennett@mymail.unisa.edu.au (M.G.A.B.); ashley.meakin@unisa.edu.au (A.S.M.); michael.wiese@unisa.edu.au (M.D.W.); 2 Department of Physiology, Development & Neuroscience, University of Cambridge, Cambridge CB2 3EG, UK; k.botting@ucl.ac.uk (K.J.B.-L.); yn252@cam.ac.uk (Y.N.); sgf27@cam.ac.uk (S.G.F.); dag26@cam.ac.uk (D.A.G.); 3 MRC Mitochondrial Biology Unit, Department of Medicine, University of Cambridge, Cambridge CB2 0XY, UK; mpm@mrc-mbu.cam.ac.uk

**Keywords:** antioxidant, antenatal corticosteroids, pregnancy, cytochrome p450, liver, offspring, health programming, preterm birth

## Abstract

**Background/Objectives**: In pregnancy threatened by preterm birth, antenatal corticosteroids (ACS) are administered to accelerate fetal lung maturation. However, they have side effects, including the production of reactive oxygen species that can impact cytochrome P450 (CYP) activity. We hypothesised that antioxidants could protect a fetus treated with ACS during gestation and prevent the programming of altered hepatic CYP activity in the offspring. The primary outcome of our study was the impact of different maternal treatments on the activity of hepatic drug-metabolising enzymes in offspring. **Methods**: At 100 ± 1 days gestational age (dGA, term = 147 dGA), 73 ewes were randomly allocated to the following: saline (5 mL IV daily 105–137 ± 2 dGA, *n* = 17), ACS (Dexamethasone (Dex); 12 mg IM at 115 and 116 dGA; *n* = 25), MitoQ (6 mg/kg MS010 IV, daily bolus 105–137 ± 2 dGA; *n* = 17) or Dex and MitoQ (Dex+MitoQ; *n* = 14). CYP activity and protein abundance were assessed using functional assays and Western blot. **Results**: Dex decreased the hepatic activity of fetal CYP3A (−56%, *P*_Dex_ = 0.0322), and 9 mo lamb CYP1A2 (−22%, *P*_Dex_ = 0.0003), CYP2B6 (−36%, *P*_Dex_ = 0.0234), CYP2C8 (−34%, *P*_Dex_ = 0.0493) and CYP2E1 (−57%, *P*_Dex_ = 0.0009). For all, except CYP1A2, activity returned to control levels with Dex+MitoQ in 9 mo lambs. In 9 mo lambs, MitoQ alone increased activity of CYP2B6 (+16%, *P*_MitoQ_ = 0.0011) and CYP3A (midazolam, +25%, *P*_MitoQ_ = 0.0162) and increased CAT expression (*P*_MitoQ_ = 0.0171). Dex+MitoQ increased CYP3A4/5 activity (testosterone, +65%, *P*_Intx_ < 0.0003), decreased CYP1A2 activity (−14%, *P*_Intx_ = 0.0036) and decreased mitochondrial abundance (*P*_Intx_ = 0.0051). All treatments decreased fetal hepatic DRP1, a regulator of mitochondrial fission (*P*_Dex_ = 0.0055, *P*_MitoQ_ = 0.0006 and *P*_Intx_ = 0.0034). **Conclusions**: Antenatal Dex reduced activity of only one CYP in the fetus but programmed the reduced activity of several hepatic CYPs in young adult offspring, and this effect was ameliorated by combination with MitoQ.

## 1. Introduction

Sixty percent of adults are overweight or obese [1], global rates of cardiovascular disease are increasing [2], and a third of adults live with multiple chronic conditions [3]. Unsurprisingly, the number of adults requiring long-term medication is increasing. Cytochrome P450 (CYP) enzymes metabolise up to 80% of all medications and have additional roles in endogenous metabolism [4]. CYP activity is impacted by many factors, including ethnicity, age, disease, the environment [4] and pregnancy [5]. These impacts can significantly alter the efficacy of drug treatment, either by reducing the concentration of a drug below therapeutic levels or by prolonging drug exposure, thereby increasing the likelihood of adverse effects. While the clinical impact of genetic polymorphisms on CYP activity is well understood [6], the impact of medications in pregnancy on CYP activity in the offspring is largely unknown.

Medication use during pregnancy has increased significantly over recent decades [7] including interventions to alleviate complications in the mother and fetus. One such intervention is the maternal administration of antenatal corticosteroids (ACS) to promote fetal lung maturation in suspected preterm birth [8]. Betamethasone is used primarily in Australia and the United States, while its stereoisomer Dexamethasone (Dex) is used in the United Kingdom. Treatment with ACS mimics the rise in fetal plasma cortisol occurring towards term, acting on the glucocorticoid receptor (GR) to promote maturation and surfactant production in the fetal lung [9,10]. Although ACS treatment reduces neonatal mortality and the incidence of respiratory distress syndrome (RDS), necrotising enterocolitis and intraventricular haemorrhage [11,12], there is emerging research highlighting potential long-term side effects, including an increased risk of cognitive disorders in childhood and increased blood pressure in early adolescence [13,14,15]. However, the long-term consequences of ACS during pregnancy for drug metabolism and clearance in the offspring are completely unknown.

Glucocorticoids promote excess reactive oxygen species (ROS) generation, and ACS induces persistent oxidative stress in newborn offspring, resulting in further impacts in several organs and systems [16,17,18,19]. Therefore, we have previously hypothesised that glucocorticoid-induced oxidative stress may be a mechanism mediating the adverse effects of ACS on off-target tissue; hence, the addition of a maternal antioxidant in a pregnancy threatened by preterm birth to maintain the beneficial maturational effects of ACS on the fetal lung but limit adverse effects on non-target organs [20]. Antioxidants such as melatonin and resveratrol have demonstrated protective effects against programmed hypertension in animal studies [21,22,23]; however, results in human studies have been mixed [24,25,26]. A widespread theory to explain these subtherapeutic outcomes is that typical antioxidants do not effectively cross the mitochondrial membrane to elicit their effects [24]. Unlike melatonin and resveratrol, the antioxidant MitoQ crosses the mitochondrial membrane and accumulates at high concentrations, due to conjugation to a lipophilic triphenylphosphonium cation, to directly prevent damage through inhibiting lipid peroxidation [27]. MitoQ does not affect upstream ROS production, thereby maintaining the generation of superoxide anion necessary for cellular physiological functions [27,28]. The benefits of MitoQ against conditions of increased oxidative stress have been revealed in several studies in chicken embryos, rodents and sheep and in Phase II human trials [28,29,30,31,32,33,34,35,36]. In contrast to conventional antioxidants, MitoQ shows no adverse side effects and long-term administration in human trials for one year revealed no toxicity [34,35,36,37]. Therefore, in this study, we tested the hypothesis that ACS modulates hepatic CYP activity in fetal and adult offspring and that maternal treatment with the mitochondria-targeted antioxidant MitoQ is protective in ovine pregnancy.

## 2. Materials and Methods

All procedures were approved by the Ethical Review Committee of the University of Cambridge and complied with UK Animals (Scientific Procedures) Act 1986 under the authority of UK Home Office Project License PC6CEFE59 (granted 4 June 2019) and PP6755721 (granted 20 April 2023). The experimental design was informed by the ARRIVE guidelines [38] and samples of organs were collected in line with the 3Rs [39].

### 2.1. Surgery and Delivery of Maternal MitoQ and Dexamethasone

At 100 ± 1 days gestational age (dGA, term is ca. 147 days), 73 pregnant Welsh Mountain ewes with singleton fetuses were anaesthetised (induction: 2–3 mg kg^−1^ IV alfaxalone (Alfaxan, Jurox); maintenance: isoflurane (1.5–2% in 4:1 O_2_:N_2_O)). Surgery followed to determine the fetal sex and for the catheterisation of the maternal femoral artery and vein, as previously described [28]. Immediately prior to surgery, the ewes were administered analgesia (1.4 mg/kg SC carprofen; Rimadyl, Pfizer Ltd., Kent, UK), antibiotics (30 mg/kg IM procaine benzylpenicillin; Depocillin, Intervet UK Ltd., Milton Keynes, UK) and vitamin B12 (0.004 mg/kg IM, Anivit B12, Animal Care Ltd., York, UK). Antibiotics (30 mg/kg IM procaine benzylpenicillin; Depocillin, Intervet UK Ltd., Milton Keynes, UK) and analgesia (1.4 mg/kg SC carprofen; Rimadyl, Pfizer Ltd., Kent, UK) were administered to the ewes for 5 and 3 days, respectively, following surgery. Maternal wellbeing was monitored daily with arterial blood samples; arterial blood gas and pH were measured with an ABL5 blood gas analyser (Radiometer; Copenhagen, Denmark) and haemoglobin oxygen saturation was measured with a haemoximeter (OSM3; Radiometer; Copenhagen, Denmark). Ewes were randomly assigned to one of 4 antenatal treatment groups:

1. Saline (5 mL IV daily 105–137 ± 2 dGA, *n* = 17);
2. Dexamethasone (Dex; 12 mg in 2 mL saline IM at 115 and 116 dGA; *n* = 25);
3. MitoQ (6 mg/kg MS010 (20% *w*/*w* mixture of mitoquinol and mitoquinone, the reduced and oxidised forms of the same molecule, respectively, and β-cyclodextrin to improve solubility; MRC Mitochondrial Biology Unit, Cambridge, UK) in 5 mL saline IV, daily bolus 105–137 ± 2 dGA; *n* = 17; [28]);
4. Co-treatment with Dex and MitoQ (Dex+MitoQ (as above); *n* = 14).

The MitoQ dose was determined by the highest dose administered in human clinical trials [35] and was given as a slow bolus through the maternal venous catheter. Ewes were housed with other animals in view and had ad libitum access to food and water.

### 2.2. Post-Mortem

At 137 ± 2 dGA, ewes carrying male fetuses were humanely euthanised with an overdose of sodium pentobarbitone (0.4 mL kg^−1^ IV Pentoject; Animal Ltd., York, UK); the fetus was exteriorized by hysterotomy. Ewes carrying female fetuses were allowed to lamb naturally. Female offspring were weaned at 5 months of age (mo) and humanely killed at 9 mo with an overdose of sodium pentobarbitone (0.4 mg kg^−1^ IV Pentoject; Animal Ltd., York, UK). Liver samples were collected from offspring at both ages, snap-frozen in liquid nitrogen and stored at −80 °C until molecular analysis.

### 2.3. Microsome Extraction

Hepatic microsomes were extracted from fetal and young adult offspring liver using differential centrifugation, as previously described [40,41,42]. In brief, approximately 250 mg of frozen liver tissue was homogenised in 600 µL buffer (1.15% KCl, 1 mM EDTA, pH 7.4; Tissue Lyser, Qiagen, Switzerland). The homogenate was centrifuged (9000× *g*, 20 min, 4 °C) and the supernatant collected and centrifuged again (16,000× *g*, 60 min, 4 °C). The supernatant was then discarded, and the pellet resuspended in buffer (100 mM potassium phosphate, 20% glycerol, pH 7.4). Protein concentration was determined with a Micro Bicinchoninic Acid (BCA) assay Kit (Pierce, Thermo Fisher Scientific Inc., Rockford, IL, USA) and each sample was diluted to 4 mg/mL. Diluted microsomes were stored at −80 °C until required.

### 2.4. In Vitro Quantification of Hepatic Cytochrome P450 Activity

To determine the activity of CYP isoenzymes, hepatic microsomes were incubated with CYP-specific substrates, as previously described [40,41,43]. In brief, specific substrates for CYP1A2 (phenacetin), CYP2B6 (bupropion), CYP2C8 (amodiaquine), CYP2C19 (omeprazole), CYP2D6 (dextromethorphan), CYP1E1 (chlorzoxazone) and CYP3A (midazolam and testosterone) were individually incubated with 10–40 µg of microsomal protein in assay buffer (50 mM phosphate buffer, 2 mM magnesium chloride, pH 7.4) for a predetermined time at 37 °C. Reactions were stopped via the addition of 100% acetonitrile containing internal standards (hydroxyomepraozole-d3 and dextrorphan-d3, Toronto Research Chemicals, North York, ON, Canada) or 100% methanol and internal standard (6β-hydroxytestosterone-d3) for testosterone metabolism. Samples were centrifuged (12,000× *g*, 10 min, 4 °C), and the supernatant was transferred to a clean tube, evaporated to dryness (GeneVac EZ-2 Plus Evaporating System, GeneVac, Ipswitch, UK) and reconstituted in water. Quantitation of metabolites was determined using liquid chromatography–tandem mass spectrometry (LC-MS/MS; Shimadzu Nexera XR LC, Shimadzu, Kyoto, Japan; SCIEX 4500 Triple-Quad MS/MS, SCIEX, Framingham, MA, USA), as previously described [43]. The activity of each CYP was quantified via normalising the area under the curve (AUC) of the metabolite to the internal standard AUC, and the concentration of each analyte was determined via integration with a standard curve that ranged from 0.1 and 100 ng/mL. Enzyme activity of each sample was determined based on the amount of metabolite produced from the probe drug (pmol) per milligram of microsomal protein (mg) per minute of incubation time (i.e., pmol/mg/min).

### 2.5. Hepatic Protein Extraction

Total protein was extracted from liver tissue as previously described [44,45,46]. Briefly, liver tissue (~100 mg) was sonicated (John Morris Scientific, SA, Australia) in 1 mL lysis buffer (1 mM Tris HCL Ph 8, 5 M NaCl, 1% NP-40, 1 mM Na orthovanadate, 30 mM NaF, 10 mM Na tetrapyrophosphate and 10 mM EDTA) and a protease inhibitor tablet (complete Mini; Roche, Indianapolis, IN, USA). A Micro BCA Kit (Pierce, Thermo Fisher Scientific, Rockford, IL, USA) was used to determine the protein content of each sample using bovine serum albumin (BSA; 2 mg/mL stock solution) as a standard curve.

### 2.6. Quantification of Hepatic Proteins

Total hepatic protein was analysed by Western blot as previously described [44,45,46,47,48]. Briefly, protein samples (75 μg) were resolved using SDS-PAGE and then transferred to a nitrocellulose membrane, which was subsequently stained with PonceauS to determine the efficacy of the transfer. Membranes were cut according to the weight of the target proteins, briefly washed with 7% acetic acid to remove the PonceauS and then subjected to three 5 min washes in Tris-buffered saline (TBS). Membranes were then blocked in 5% BSA in TBS with 1% Tween-20 (TBS-T) for 1 h at room temperature, and underwent three 5 min washes in TBS-T before being incubated with their respective antibody: Anti-4 hydroxynonenal (4HNE; 1:1000, 65880, Abcam, Cambridge, UK), CAT (1:1000, D5N7V, 14097S, Cell Signaling Technology, Danvers, MA, USA), DRP1 (1:1000, 8570S, Cell Signaling Technology, Danvers, MA, USA), HNF-4α (c-19, 1:1000, sc-6556, Santa Cruz Biotechnology, Dallas, TX, USA), MitoBiogenesis Western Blot Cocktail (1:1000, ab123545, Abcam, Cambrdge, UK), Mitofusion-2 (1:1000, 80417S, Cell Signaling Technology, Danvers, MA, USA), OPA1 (1:1000, 804471S, Cell Signaling Technology, Danvers, MA, USA), PPARα (1:1000, sc-9000, Santa Cruz Biotechnology, Dallas, TX, USA) or SOD (1:1000, #06-984, Merck, Darmstadt, Germany). After incubation with the primary antibody, the blots were washed and incubated with the appropriate HRP-labelled secondary IgG antibody for 1 h at room temperature. SuperSignal West Pico Chemiluminescent Substrate (Thermo Scientific, Waltham, MA, USA) was used to detect reactive bands by enhanced chemiluminescence. Western blots were imaged using ImageQuant LAS4000 (GE Healthcare, Chicago, IL, USA), and protein abundance was quantified by densitometry using Image Quant software (version 8.1, GE Healthcare, Chicago, IL, USA) and normalised to β-actin (β-actin Peroxidase, 1:10,000, A3854, Sigma-Aldrich, St. Louis, MO, USA) or Vinculin (Vinculin (E1E9V) XP Rabbit mAb (HRP Conjugate), 1:2000, 18799S, Cell Signaling Technology, Danvers, MA, USA). When appropriate, samples were excluded due to unanalysable bands, denoted by an ‘X’ overlaying the band.

### 2.7. Statistical Analysis

All statistical analyses were performed using GraphPad Prism 8 (GraphPad Software, Inc., La Jolla, CA, USA). Outliers were identified with Grubbs’ analysis and excluded if α < 0.05. If the activity of a sample was below the activity of the negative controls (i.e., no microsomes or no NADPH), it was excluded from analysis. Data are presented as mean ± SEM and analysed using two-way ANOVA (factors = Dex and MitoQ) with the Tukey post hoc test. Data normality was checked with the Shapiro–Wilk test, and non-normally distributed data were ln-transformed prior to statistical analysis. Statistical results are shown on non-transformed data, and *p* < 0.05 was considered significant for all analyses.

## 3. Results

### 3.1. Antenatal MitoQ Increases Liver Weight in the Fetus and Increases Bodyweight in 9 mo Lambs

Maternal treatment with MitoQ increased bodyweight and liver weight in fetuses; however, when combined with Dex, liver weight was not changed by MitoQ treatment (Table 1). Conversely, Dex decreased fetal liver to bodyweight ratio. In young adult lambs, post-mortem bodyweight was increased by antenatal MitoQ, while only combined treatment with Dex and MitoQ increased liver weight (Table 1).

### 3.2. Metabolism of Substrates in the CYP3A Family Is Altered in Fetuses and Young Adult Offspring According to Antenatal Treatment

**Figure 1:**
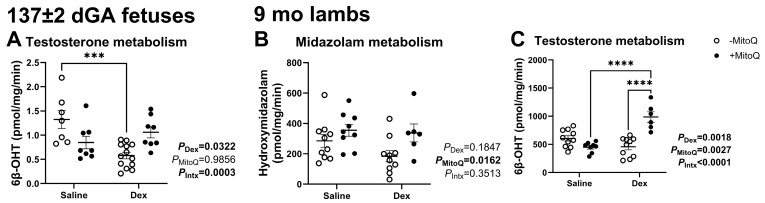
Hepatic CYP3A activity is impacted by antenatal treatments in late gestation in fetal and young adult offspring. Fetal in vitro hepatic metabolism of testosterone to (**A**) 6β-hydroxytestosterone (6β-OHT). In vitro hepatic metabolism in 9 mo lambs: (**B**) midazolam to hydroxymidazolam and (**C**) testosterone to 6β-OHT. *** *p* < 0.001, **** *p* < 0.0001.

Consistent with our previous work, CYP3A metabolism of midazolam to hydroxymidazolam was not measurable in hepatic microsomes from 137 ± 2 dGA fetuses (42). However, CYP3A-mediated metabolism of testosterone to 6β-hydroxytestosterone (6β-OHT) was detectable in the fetus, and activity was significantly reduced by maternal Dex (*P*_Dex_ = 0.0322; Figure 1A), but only in the absence of MitoQ (*P*_intx_ = 0.0003; Figure 1A). In 9 mo lambs exposed to MitoQ in utero, hepatic CYP3A metabolism of midazolam was increased (*P*_MitoQ_ = 0.0162; Figure 1B). CYP3A metabolism of testosterone to 6β-OHT was increased due to MitoQ (*P*_MitoQ_ = 0.0027) and Dex (*P*_Dex_ = 0.0018), but significance was driven by combined treatment (*P*_Intx_ < 0.0001; Figure 1C).

### 3.3. Decreased Hepatic CYP Activity in Young Adult Lambs as a Result of Maternal Dexamethasone Treatment in Pregnancy Can Be Ameliorated by MitoQ Co-Treatment

**Figure 2:**
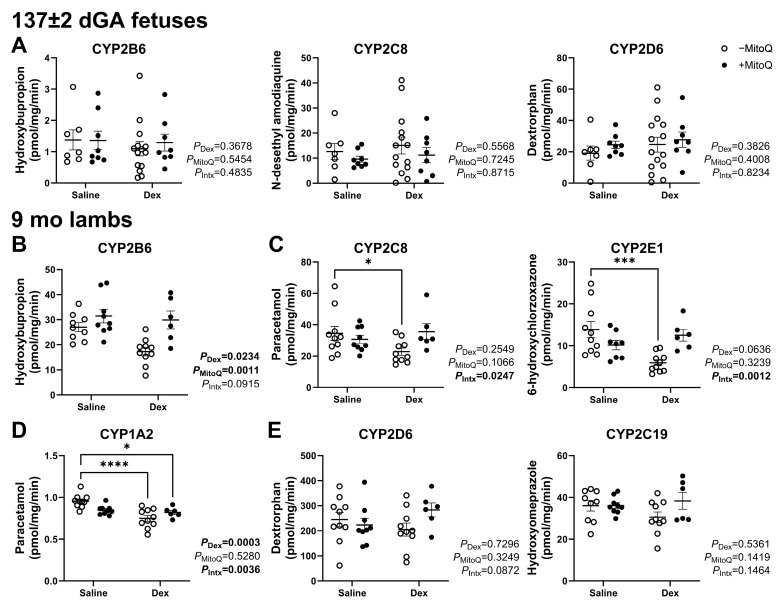
Antenatal treatments in late gestation impact young adult but not fetal hepatic CYP activity. In vitro fetal hepatic (**A**) CYP2B6, CYP2C8 and CYP2D6 activity. In vitro young adult hepatic (**B**) CYP2B6, (**C**) CYP2C8, CYP2E1, and (**D**) CYP1A2, (**E**) CYP2D6 and CYP2C19 activity. * *p* < 0.05, *** *p* < 0.001, **** *p* < 0.0001.

Antenatal treatments did not alter hepatic CYP2B6, CYP2C8 or CYP2D6 activity in the 137 ± 2 dGA fetus (Figure 2A). Activity in CYP1A2, CYP2E1 and CYP2C19 was not detectable at this gestational age (43). CYP2B6 activity in 9 mo lambs was decreased by Dex treatment (*P*_Dex_ = 0.0234) and normalised by maternal MitoQ treatment (*P*_MitoQ_ = 0.0011; Figure 2B). Post hoc analysis showed that CYP2C8 and CYP2E1 activity was decreased in lambs exposed to Dex only (*P*_Intx_ = 0.0247 and 0.0012 respectively; Figure 2C), while CYP1A2 activity was decreased by Dex exposure both with and without MitoQ (*P*_Dex_ = 0.0003; Figure 2D). CYP2D6 and CYP2C19 were not impacted in 9 mo lambs (Figure 2E).

### 3.4. Antenatal MitoQ Increases Endogenous Antioxidant Catalase Expression in 9 mo Lambs

**Figure 3:**
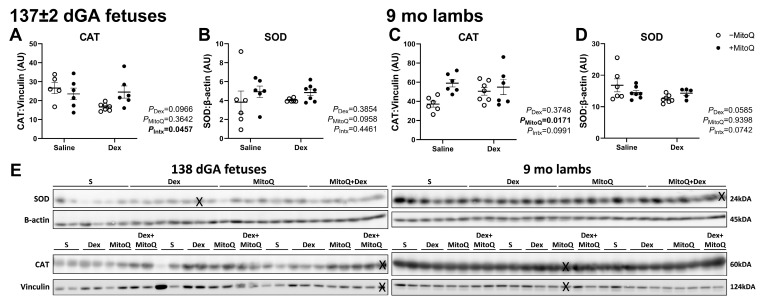
Relative protein abundance of endogenous antioxidants: (**A**,**C**) catalase (CAT) and (**B**,**D**) superoxide dismutase (SOD). Protein normalised to β-actin or Vinculin. Each data point represents an individual animal. An ‘X’ is superimposed over excluded lanes (**E**).

Fetal catalase (CAT) and superoxide dismutase (SOD) protein expression were not impacted by Dex or MitoQ (Figure 3A,B). CAT protein was increased in MitoQ-exposed lambs at 9 mo (*P*_MitoQ_ = 0.0171; Figure 3C). SOD was not altered by any antenatal treatments in 9 mo lambs (Figure 3D).

### 3.5. Mitochondrial Abundance Is Decreased in 9 mo Lambs, but Not Fetuses, Co-Treated with Dex+MitoQ

**Figure 4:**
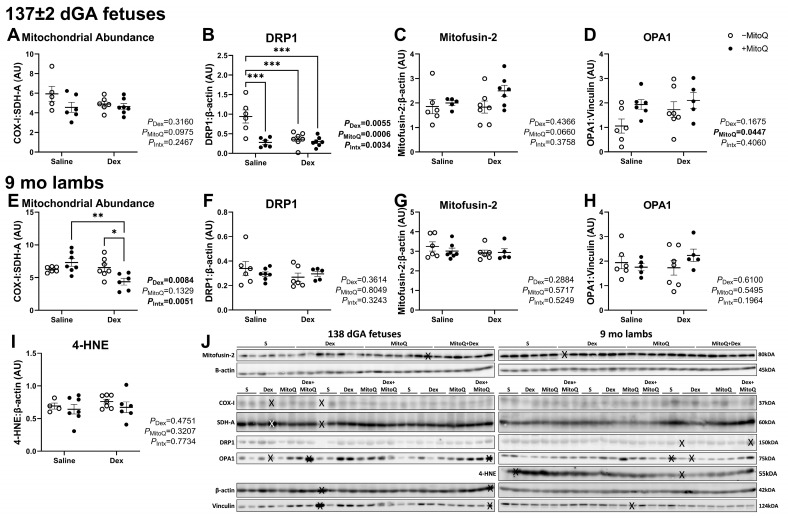
Markers of mitochondrial function and abundance are impacted by antenatal treatments in fetal and young adult offspring. (**A**,**E**) Mitochondrial abundance as represented by a ratio of mitochondrial-derived COX-I to nuclear-derived SDH-A, (**B**,**F**) dynamin-related protein 1 (DRP1), (**C**,**G**) mitofusion-2, (**D**,**H**) mitochondrial dynamin-like GTPase (OPA1) and (**I**) 4-hydroxynonenal (4HNE). Protein normalised to β-actin or Vinculin. Each data point represents an individual animal. An ‘X’ is superimposed over excluded lanes (**J**). * *p* < 0.05, ** *p* < 0.01, *** *p* < 0.001.

Fetal mitochondrial abundance did not differ between antenatal treatment groups (Figure 4A), although the protein expression of dynamin-related protein 1 (DRP1) was decreased in fetuses in all groups compared to controls (Figure 4B). Conversely, MitoQ treatment increased mitochondrial dynamin-like GTPase (OPA1) protein expression (Figure 4D). Antenatal Dex and MitoQ co-treatment decreased mitochondrial abundance in 9 mo lambs compared to controls (Tukey’s post hoc *P* = 0.0287; Figure 4E). Antenatal treatments did not alter the protein expression of DRP1, MitoFusin-2 and OPA1 (Figure 4C,F,H). 4-Hydroxynonenal was not expressed in fetuses (Supplementary Material with original western blot images) and did not change with any treatment in the 9 mo lambs (Figure 4I).

### 3.6. CYP Transcription Regulators Were Not Affected by Antenatal Treatments

**Figure 5:**
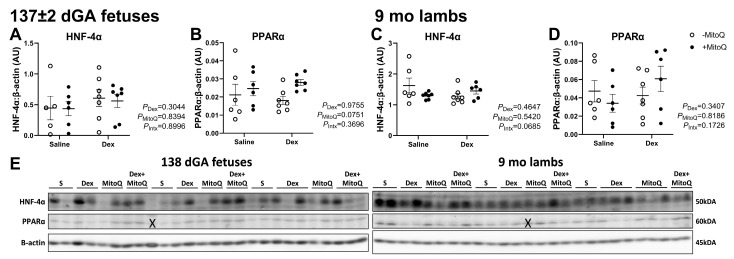
Transcription regulators of CYPs, HNF-4α and PPARα were unaffected by antenatal treatments in both fetuses and lambs. (**A**,**C**) Hepatocyte nuclear factor 4 alpha (HNF-4α) and (**B**,**D**) peroxisome proliferator-activated receptor alpha (PPARα). Protein normalised to β-actin. Each data point represents an individual animal. An ‘X’ is superimposed over excluded lanes (**E**).

The protein expression of both HNF-4α and PPARα was not altered by antenatal treatment in fetuses (Figure 5A,B) or 9 mo lambs (Figure 5C,D).

## 4. Discussion

In this manuscript, we show that the hepatic activity of CYP1A2, CYP2B6, CYP2C8 and CYP2E1 was decreased in 9-month-old offspring exposed to Dex in utero due to maternal ACS treatment in healthy sheep pregnancy. The sustained impact on CYP activity suggests that this is a programmed effect, and that despite being born at term, the metabolism of many medications taken over a lifetime may be affected in these offspring. We also show that MitoQ can protect against the Dex-induced developmental programming of decreased CYP activity for CYP2B6, CYP2C8 and CYP2E1 in normal pregnancy in sheep. However, CYP1A2 activity was decreased with MitoQ alone or co-treatment with Dex. Conversely, Dex treatment decreased CYP3A activity in the fetus, while Dex+MitoQ treatment increased CYP3A activity in young adult offspring.

In the present study, the CYPs affected by Dex, namely CYP1A2, CYP2B6, CYP2C8 and CYP3A, metabolise more than half of clinically used medications, including those used for the treatment of conditions linked with complicated pregnancies [4]. For example, CYP1A2 metabolises caffeine, analgesics (paracetamol and lignocaine), antidepressants (amitriptyline and duloxetine) and antipsychotics (clozapine and olanzapine) [4,49]. CYP1A2 is also involved in the metabolism of endogenous substrates, such as arachidonic acid, prostaglandins and oestrogens [4]. CYP2B6 metabolises anaesthetics (propofol and ketamine), the µ-opioid agonist methadone and the chemotherapy drug cyclophosphamide [50]. Many of these drugs have a narrow therapeutic window, and patients homozygous for a CYB2B6 loss-of-function genetic variant show a significant risk of adverse drug reactions and poorer outcomes [51,52]. Therefore, combining past and present data highlights that maternal ACS treatment may program an increased risk of adverse drug reactions and poor outcomes in later life, independent of individual genotype, by affecting hepatic CYP2B6 activity in the offspring.

In humans, midazolam and testosterone are both metabolised by members of the CYP3A family, but we cannot determine which members of this family are responsible for the formation of specific metabolites due to their high heterogeneity. Despite differences in the active sites impacting drug binding and efficacy, separating the metabolic responsibilities of CYP3A4/5 and the fetal isoform CYP3A7 is currently not possible. Although midazolam and testosterone are both validated CYP3A substrates in sheep and other large animals [53], only the CYP3 A24 isoform is currently annotated for sheep [54]. This may explain the different outcomes for CYP3A activity depending on the substrate used or may represent differences in binding affinity for these substrates. As Dex is an inducer of CYP3A [55], the decrease in fetal CYP3A activity due to Dex treatment may be due to the timing of administration in this study or via ACS-induced ROS. Likewise, inflammatory markers, including c-reactive protein and interleukin-6, downregulate CYP3A activity, [56] and both of these markers are associated with oxidative stress [57]: a potential explanation for the short-term effects on fetal CYP3A activity due to ACS-induced ROS. Dex has a half-life of 4 h [58], and in animal models it is undetectable 24 h after fetal infusion has ceased [59,60]. In this study, samples were collected more than 20 days after ACS administration, leaving a considerable wash-out period, thus potentially explaining the lack of acute impact on most fetal CYPs measured.

Although CYP1A2, CYP2B6, CYP2C8 and CYP3A activity was impacted by Dex, CYP2C19 activity was not, despite the 80% genetic similarity to CYP2C8 [61]. The regulatory mechanisms and genetic sequences for CYPs overlap significantly, particularly for members of the CYP2C family, but we did not observe a consistent impact within this family. Despite the similarity in regulation and genetics, CYP2C8 and CYP2C19 have a relatively well defined separation of substrates; CYP2C8 substrates include relatively large and weakly acid drugs, while CYP2C19 substrates are typically neutral or weakly basic [4]. HNF-4α is a regulatory protein for CYP2C8, CYP2C9, CYP2D6 and CYP3A but not CYP2C19, despite similar HNF-4α binding sites on the promotor of CYP2C9 [4]. Therefore, we investigated whether HNF-4α is a possible effector for the changes in activity measured in young adult lambs. However, its expression was not affected by maternal treatments, suggesting epigenetic programming occurring directly to CYP genes and/or post-translational impacts in affected CYP enzymes. We also investigated PPARα expression, as it regulates CYP3A4 in human hepatocytes, and it is involved in inflammatory pathways; however, we observed no change in hepatic PPARα expression in fetal or adult offspring.

Fetal adaptations in pregnancy are sex-specific [62], and so may be the programming of CYP activity [4]. Female and male placentae express different GR isoform profiles [63]. Dex acts on the GR, and the dimorphic adaptation of expression between sexes may contribute to long-term programming. Additionally, fetal sex impacts how the pregnancy reacts to environmental changes in utero and in the neonatal period [62,64]. Females are reported to adapt better to adverse impacts in pregnancy, whereas males appear to prioritise growth and therefore have an increased risk of late-gestation complications and perinatal mortality [65]. Females born within 72 h of ACS exposure have increased plasma cortisol concentrations, despite the placental activity of 11B-HSD2 being greater than in male pregnancies at this timepoint [66]. Hepatic CYP2B6 and CYP3A activity was affected by maternal obesity in males but not females, and this was associated with differences in GR isoform profiles [67]. Therefore, it is possible that the fetal and young adult outcomes in this study were impacted by sex, which may explain the differences in alterations in CYP activity between timepoints in this study. However, a limitation of the current study is that it was not designed to determine sexually dimorphic effects, as males were studied in fetal life and females in young adulthood. This ensured every singleton pregnancy was used, and ewe rather than ram lambs were kept until adulthood as they are easier to house. Thus, future studies could include both male and female offspring.

Dex+MitoQ decreased a marker of oxidative stress and mitochondrial abundance in the liver of young adult but not fetal offspring. The presence and regulation of DRP1 is essential for survival [68], and it protects against liver disease by preventing endoplasmic reticulum (ER) stress and reducing inflammatory responses [69]. The observed decrease in mitochondrial abundance in young adult lambs may be due to ER stress and decreased DRP1 in the fetus. As females respond better than males to stress during gestation [62], the impact of antenatal treatment may be larger in males than females, explaining the finding that all treated groups had decreased DRP1 in the male fetus but only Dex+MitoQ decreased mitochondrial abundance in female young adults. As the mitochondria and ER are linked for cellular homeostasis, the correct function of each organelle is reliant on the other [70]. More recently, the interaction between the mitochondria and ER has been investigated in Alzheimer’s, type 2 diabetes, obesity and myocardial infarction [71,72,73,74]. These data suggest that increased oxidative stress correlates with ER stress. Similar findings of an interaction between ER and oxidative stress have been reported in placenta isolated from sheep exposed to hypoxic pregnancy [75,76]. Therefore, MitoQ, through protecting against mitochondria-derived ROS, may decrease ER stress [75,76,77]. The prevention of ER stress by MitoQ could be a mechanism by which MitoQ ameliorates the Dex-induced programming of altered hepatic CYP activity.

ACS administration is routinely used in threatened preterm labour, and although it is indispensable for survival in babies born at 24 to 26 weeks gestation, approximately 50% of women administered ACS deliver after 35 weeks of gestation, when ACS no longer provide additional benefit for the maturation of the baby’s lungs [78,79,80,81,82]. Negative outcomes due to ACS exposure have recently become a concern, due to evidence of exposure increasing the risk of neurodevelopmental impacts in early childhood [14] and cardiovascular dysfunction in offspring [10,83]. We previously showed that in fetal and neonatal sheep, there is a decrease in CYP3A activity due to chronic placental restriction [40,84], suggesting CYP activity may also be impacted by epigenetic changes during intrauterine development. The data in the present study add to this body of evidence, suggesting that medications may be metabolised differently in young adults born at term from a healthy pregnancy that received ACS. This may lead to poorer outcomes from therapeutic treatments during their lifetime, secondary to either sub-therapeutic dosing and/or impaired drug metabolism prolonging drug exposure.

## 5. Conclusions

Antenatal Dex affected the activity of only one hepatic CYP in the fetus, but it programmed a reduction in activity of most hepatic CYPs in young adult offspring. The latter effect was ameliorated by the addition of antenatal MitoQ. Therefore, these results suggest that young adults born from clinically normal term pregnancies whose mothers received ACS treatment may have altered metabolism of medications, leading to either sub-therapeutic dosing or increased off-target effects. Further, maternal antenatal treatment with a mitochondria-targeted antioxidant appears protective against these potential ACS-induced effects in offspring. However, investigating the potential for sex differences in the long-term programming of offspring hepatic CYP activity by ACS is required. In addition, understanding the potential additive effect of Dex in a pregnancy complicated by preterm birth, chronic fetal hypoxia and/or fetal growth restriction on programmed offspring hepatic CYP function also warrants further investigation.

## 6. Patents

Michael P. Murphy had patent #6,331,532 (expired 2018) issued to Michael P. Murphy and Robin A.J. Smith for the molecular design of MitoQ.

## Figures and Tables

**Table 1:** Impact of antenatal treatments on fetal and young adult offspring characteristics.

		Saline		Dex				
		−MitoQ	+MitoQ	−MitoQ	+MitoQ	*P*_Dex_	*P*_MitoQ_	*P*_Intx_
**Fetal** **(137 ± 2 dGA)**		*n* = 7	*n* = 8	*n* = 10	*n* = 8			
	**Post-mortem bodyweight** **(kg)**	3.34 ± 0.20	4.11 ± 0.26	3.73 ± 0.16	3.82 ± 0.15	0.7989	**0.0395**	0.1013
	**Liver weight** **(g)**	68.26 ± 4.82	97.36 ± 5.20	75.38 ± 4.75	75.28 ± 2.80	0.1762	**0.0115**	0.0110
	**Liver to bodyweight ratio** **(g/kg)**	20.44 ± 0.79	24.01 ± 1.32	20.22 ± 1.09	19.12 ± 0.62	**0.0461**	0.3232	0.0672
**Young adults** **(9 mo)**		*n* = 10	*n* = 9	*n* = 10	*n* = 6			
	**Gestational age at birth** **(dGA)**	147.20 ± 0.63	146.89 ± 0.84	146.90 ± 1.09	148.00 ± 1.10	0.6692	0.6777	0.4586
	**Birthweight** **(kg)**	3.36 ± 0.21	3.34 ± 0.21	3.58 ± 0.29	3.63 ± 0.30	0.3291	0.9435	0.8788
	**Post-mortem bodyweight** **(kg)**	25.70 ± 1.51	28.44 ± 1.83	26.55 ± 1.86	34.35 ± 2.08	0.0770	**0.0076**	0.1808
	**Liver weight** **(g)**	387.39 ± 19.61	374.74 ± 21.56	374.76 ± 18.48	452.59 ± 23.60	0.1324	0.1326	**0.0400**
	**Liver to bodyweight ratio** **(g/kg)**	14.51 ± 0.63	13.30 ± 0.51	14.73 ± 1.20	13.25 ± 0.51	0.9247	0.1318	0.8816

## Data Availability

The data generated and analysed during this study are available from the corresponding author on reasonable request.

## Supplementary Materials

The following supporting information can be downloaded at: https://www.mdpi.com/article/10.3390/pharmaceutics17030285/s1, Figure S1: uncropped Fetal (upper) and Lamb (lower) western blots.

## Abbreviations

The following abbreviations are used in this manuscript:

11β-HSD2	11-beta-hydroxysteroid dehydrogenase 2
4HNE	4-hydroxynonenal
ACS	Antenatal corticosteroids
CAT	Catalase
CYP	Cytochrome P450
Dex	Dexamethasone
dGA	Days gestational age
DRP1	Dynamin-related protein 1
ER	Endoplasmic reticulum
GR	Glucocorticoid receptor
HNF-4α	Hepatocyte nuclear factor 4 alpha
IM	Intramuscular
IV	Intravenous
mo	Months of age
NADPH	Nicotinamide adenine dinucleotide phosphate
OPA1	Mitochondrial dynamin-like GTPase 1
PPARα	Peroxisome proliferator-activated receptor alpha
RDS	Respiratory Distress Syndrome
ROS	Reactive oxygen species
SEM	Standard error of the mean
SOD	Superoxide dismutase
