# Supplementary material for: Maternal MitoQ Treatment Is Protective Against Programmed Alterations in CYP Activity Due to Antenatal Dexamethasone
Source: Pharmaceutics. 2025 Feb 22;17(3):285. doi: 10.3390/pharmaceutics17030285 (PMC11944367; doi:10.3390/pharmaceutics17030285)

Supplementary figures: uncropped western blots

1st band from the left is a pooled (control) sample

Figure 3. Relative protein abundance of oxidative stress markers.

S1: SOD: Fetal (upper) and Lamb (lower)

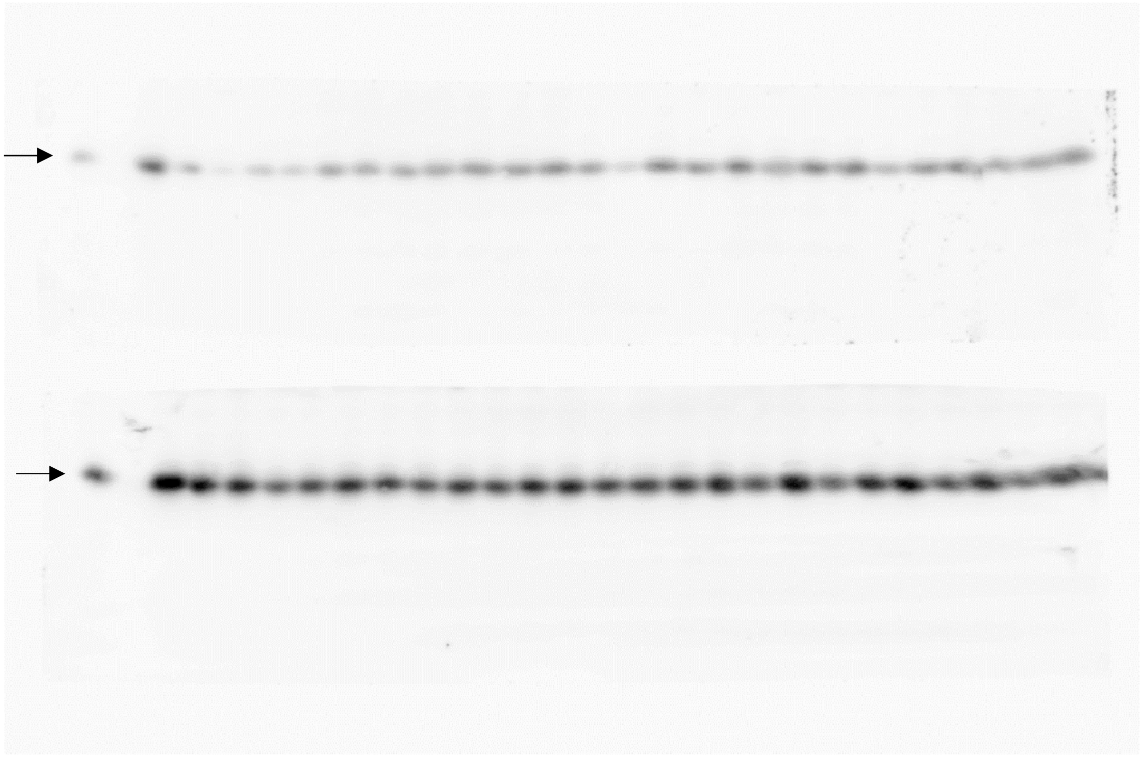

S2: B-actin: Fetal (upper) and Lamb (lower)

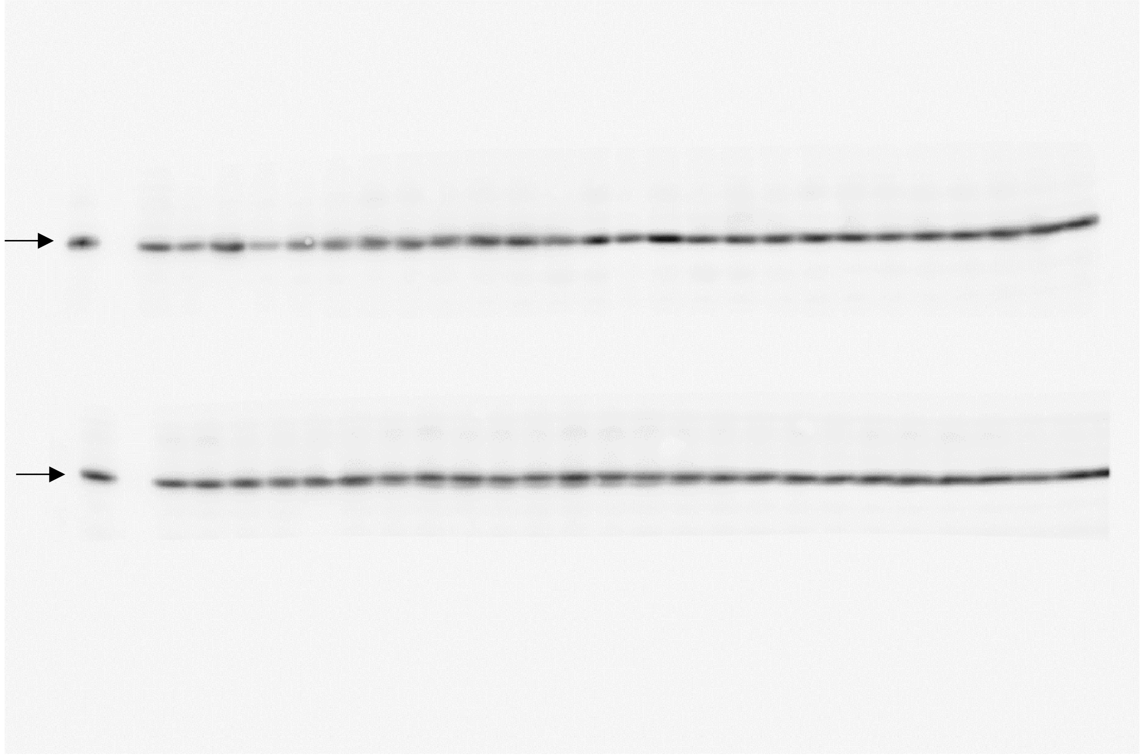

S3: CAT: Fetal (upper) and Lamb (lower)

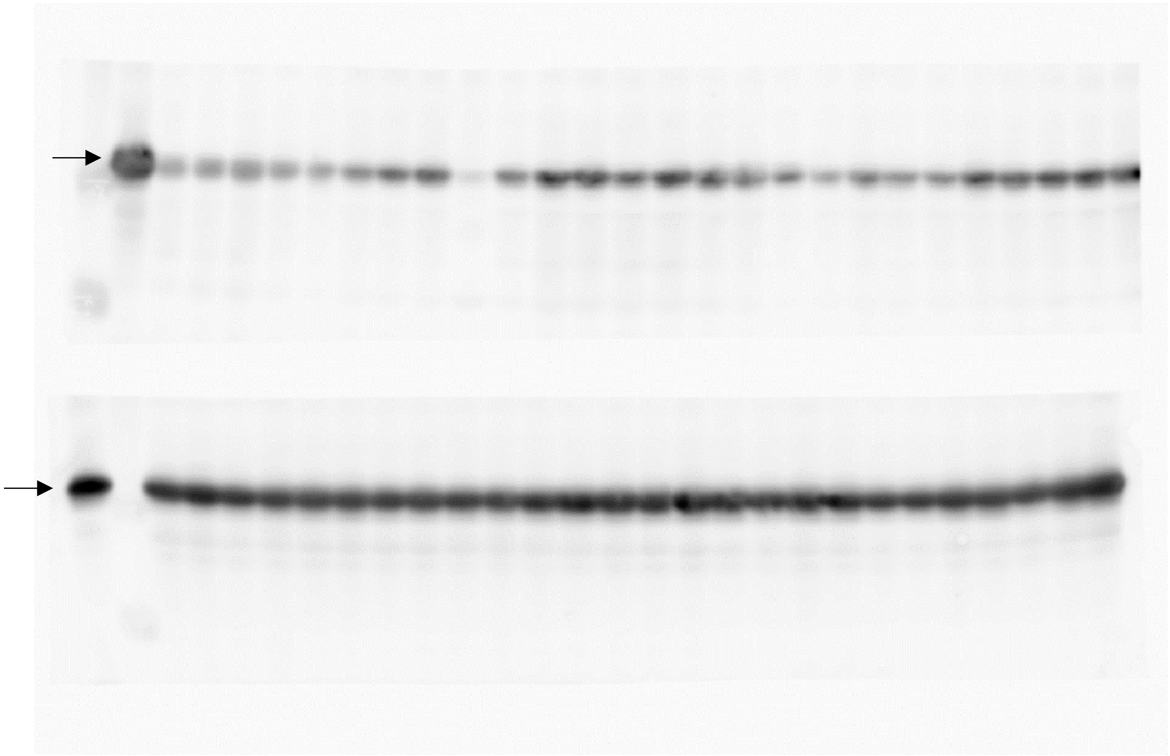

S4: Vinculin: Fetal (upper) and Lamb (lower)

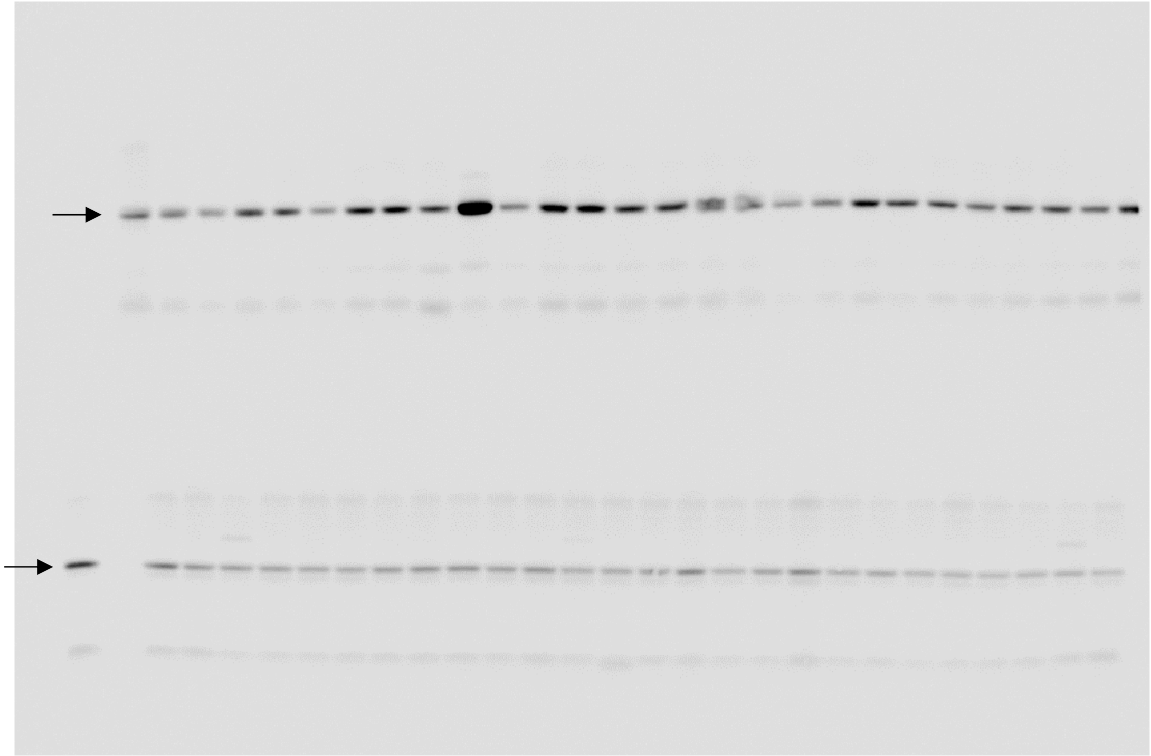

Figure 4. Markers of mitochondrial function and abundance are impacted by antenatal treatments in fetal and young adult offspring.

S5: Mitofusin-2: Fetal (upper) and Lamb (lower)

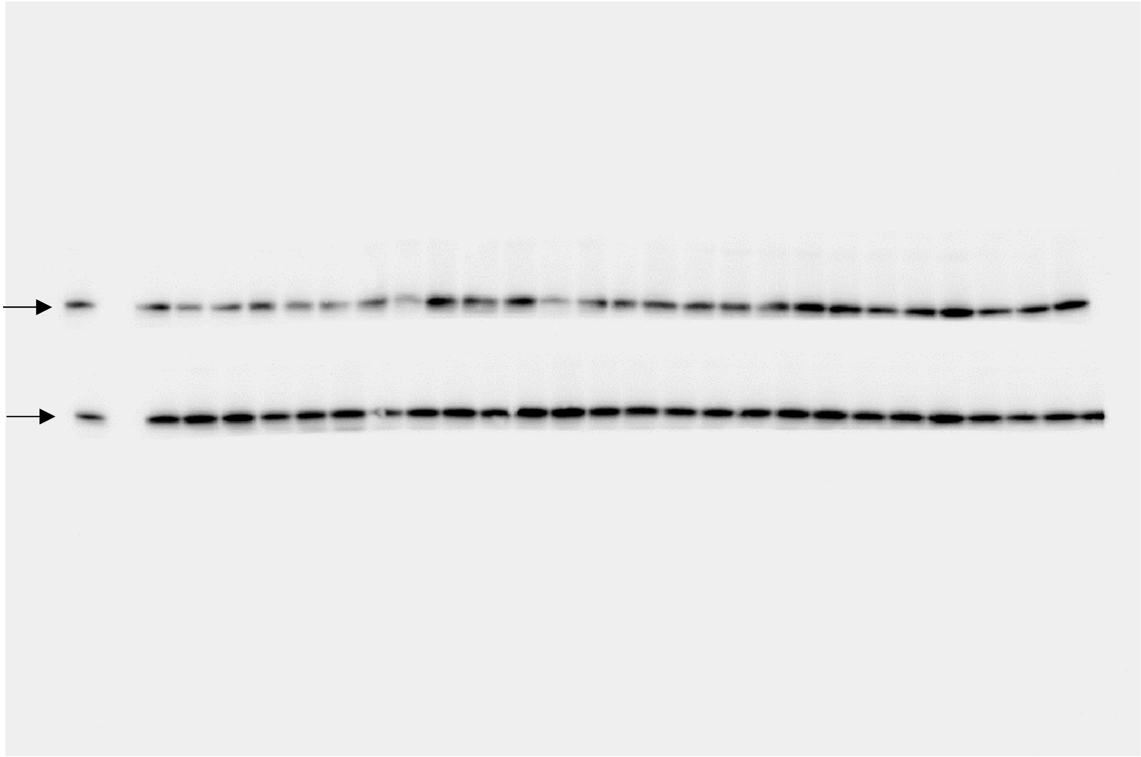

S6: B-actin: Fetal (upper) and Lamb (lower)

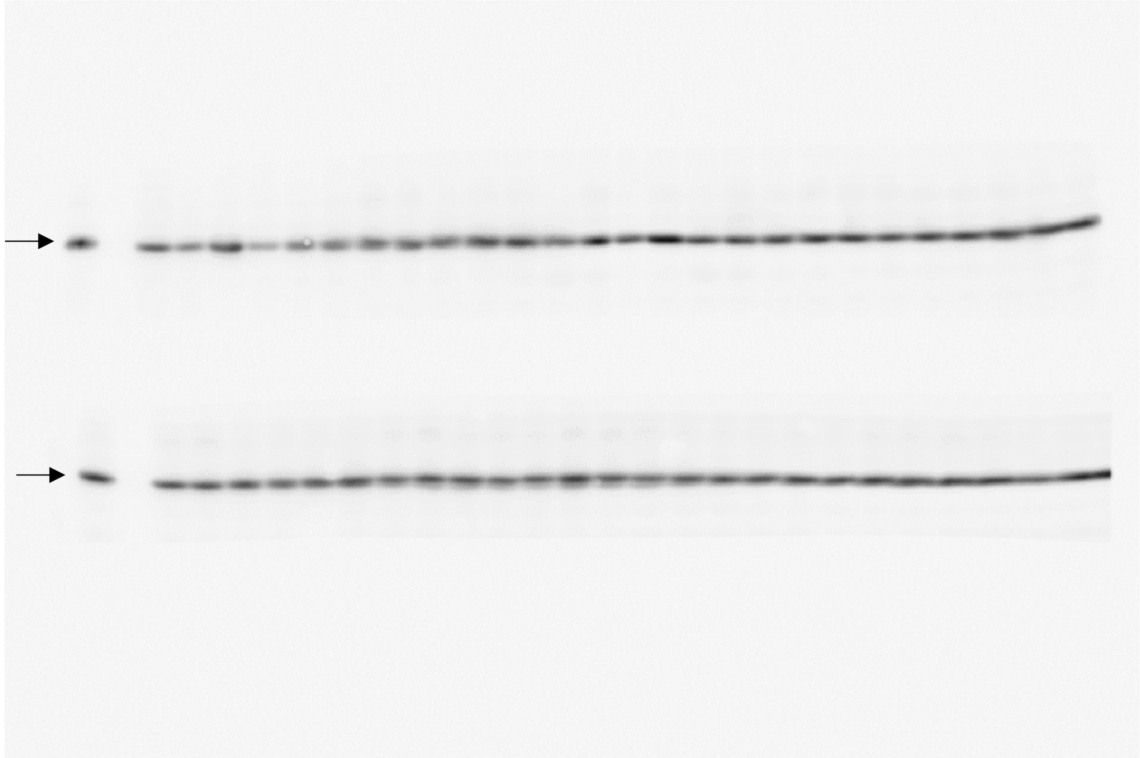

S7: COX-I: Fetal (upper) and Lamb (lower)

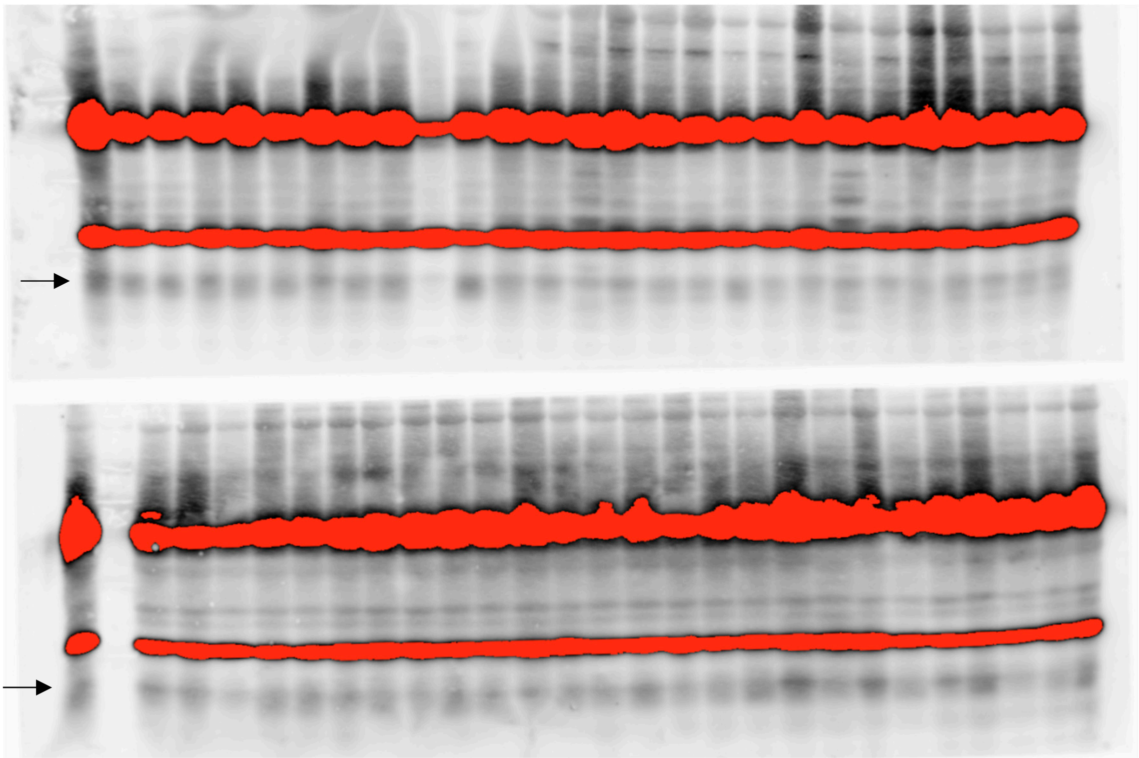

S8: SDH-A: Fetal (upper) and Lamb (lower)

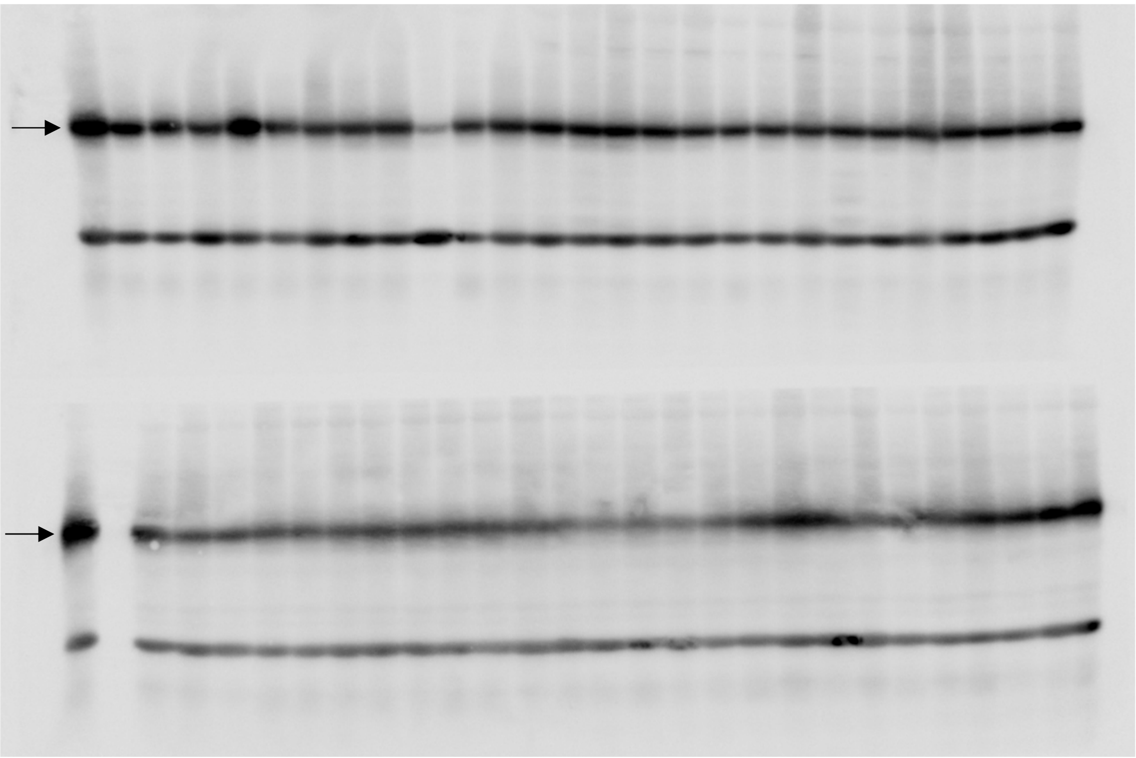

S9: DRP1: Fetal (upper) and Lamb (lower)

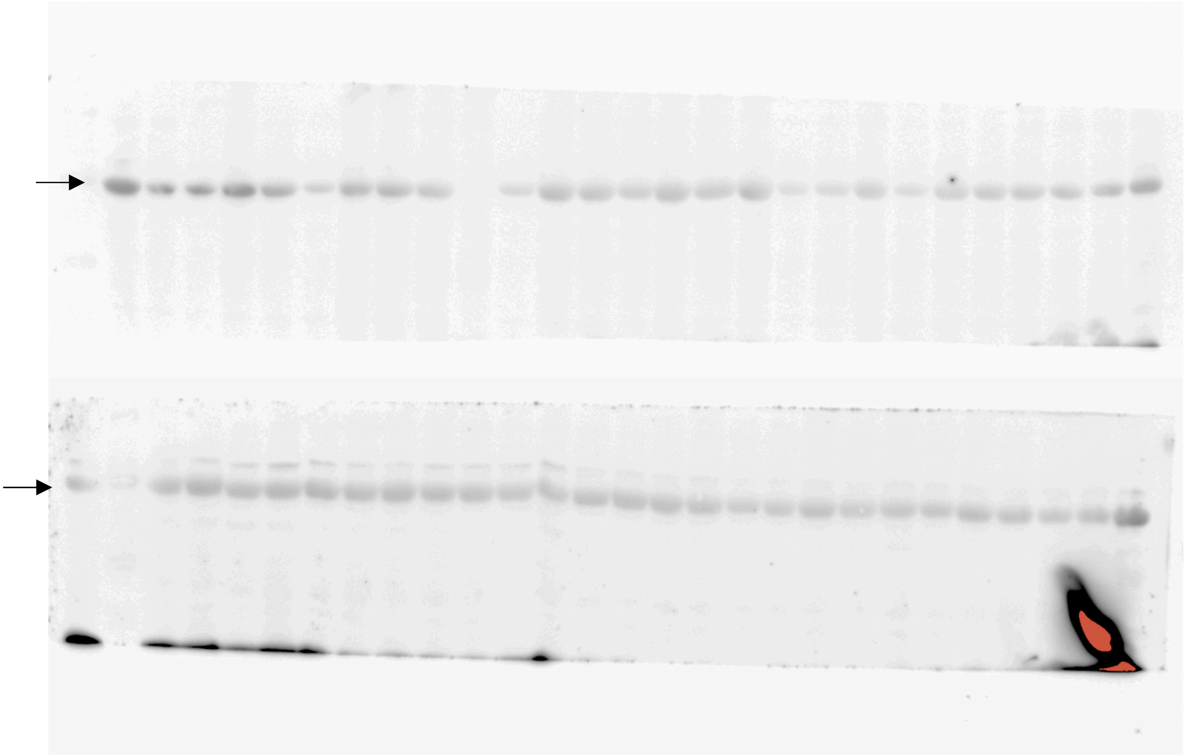

S10: OPA1: Fetal (upper) and Lamb (lower)

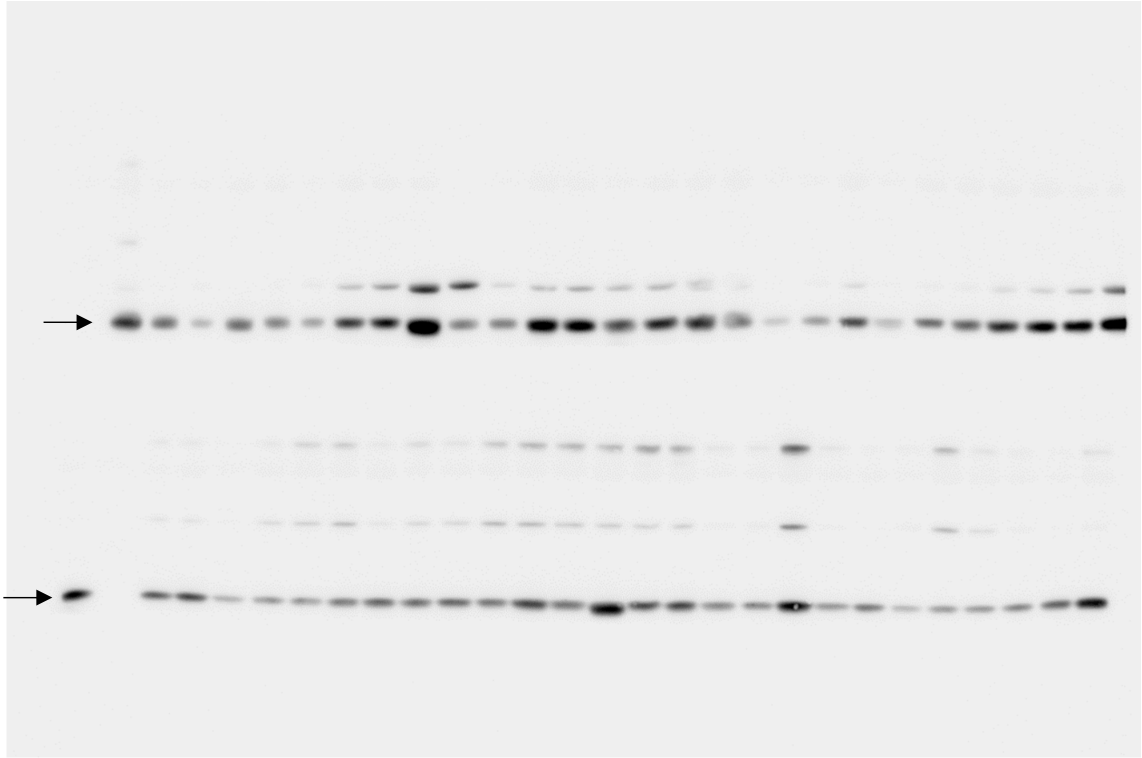

S11: 4-HNE Fetal (upper) and Lamb (lower) western blot. No fetal band at same weight as pooled sample so fetal blot not analysed.

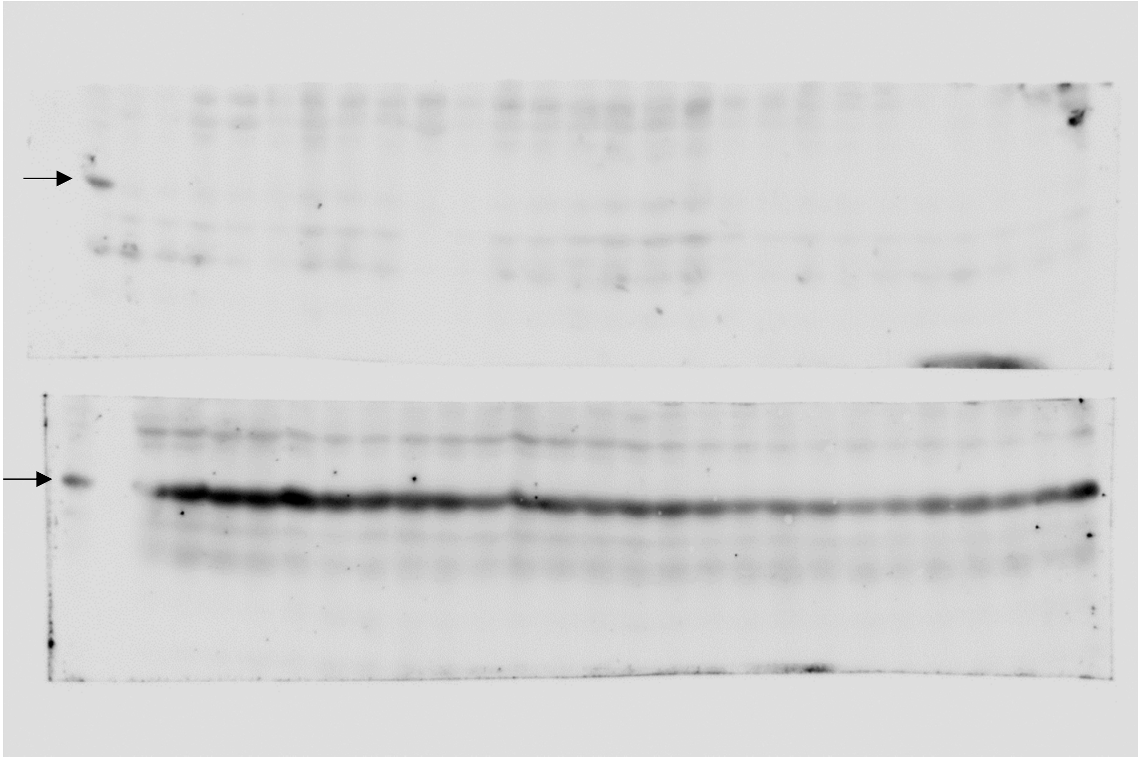

S12: B-actin: Fetal (upper) and Lamb (lower)

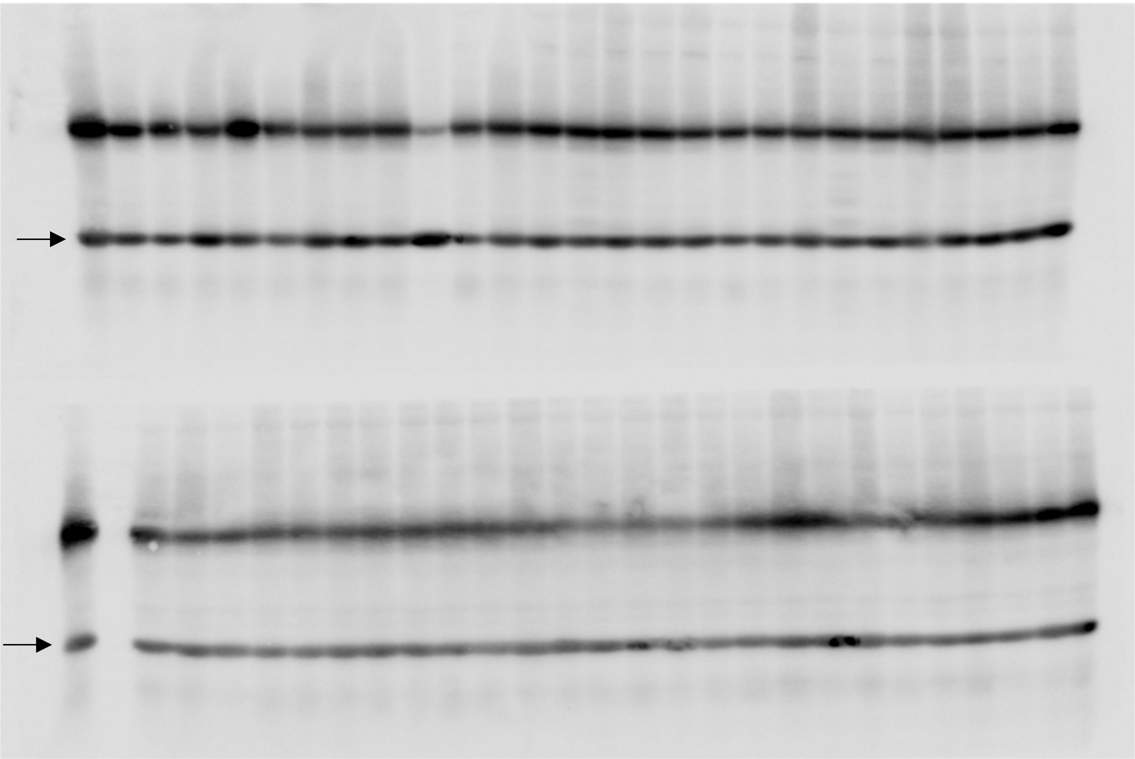

S13: Vinculin: Fetal (upper) and Lamb (lower)

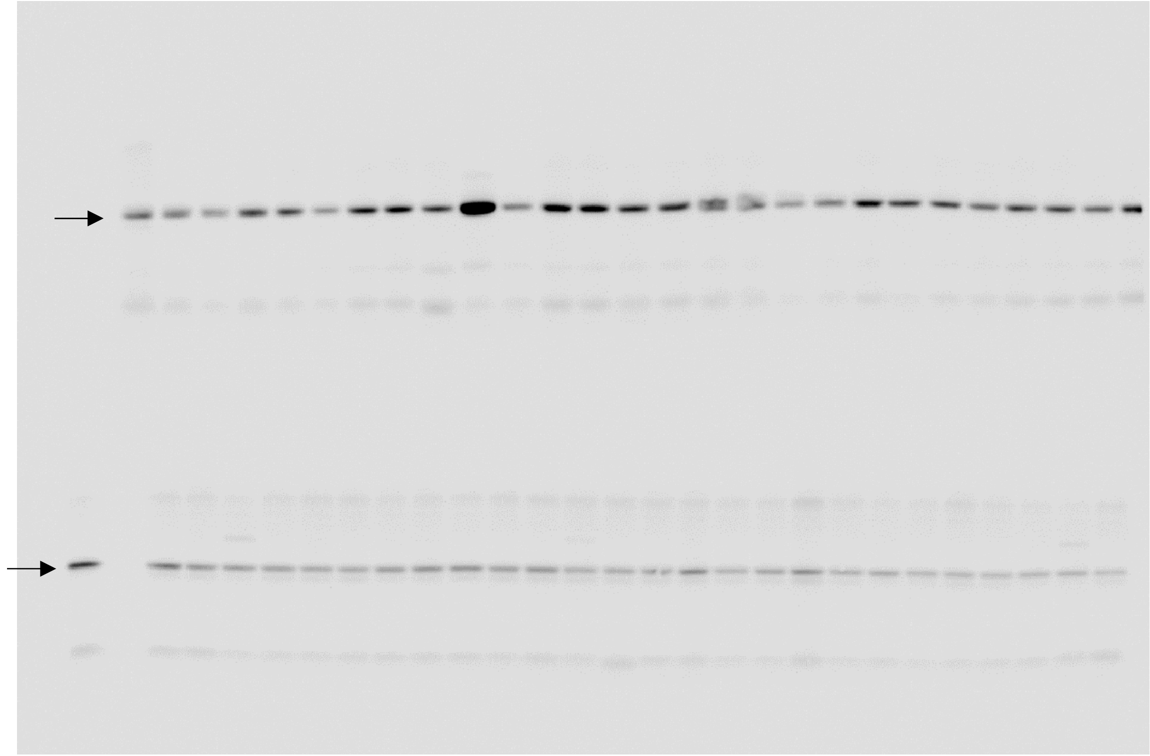

Figure 5. Transcription regulators of CYPs, HNF-4α and PPARα, were unaffected by antenatal treatments in both fetuses and lambs.

S14: HNF-4a: Fetal (upper) and Lamb (lower)

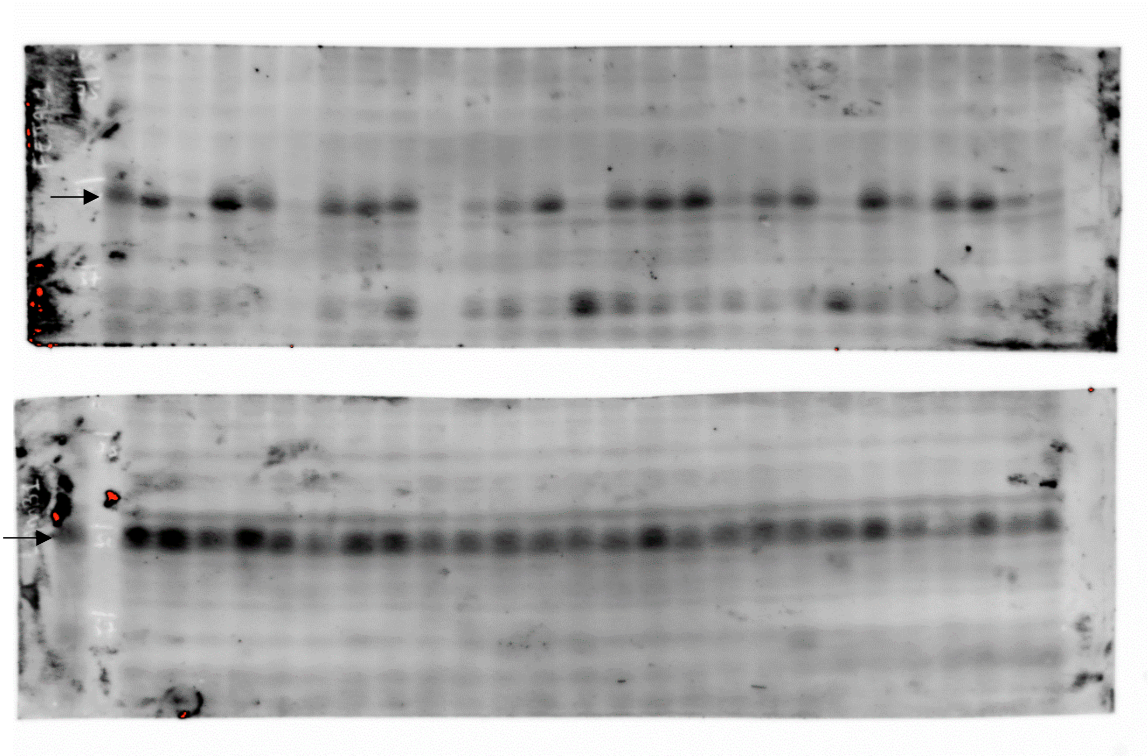

S15: PPARa: Fetal (upper) and Lamb (lower)

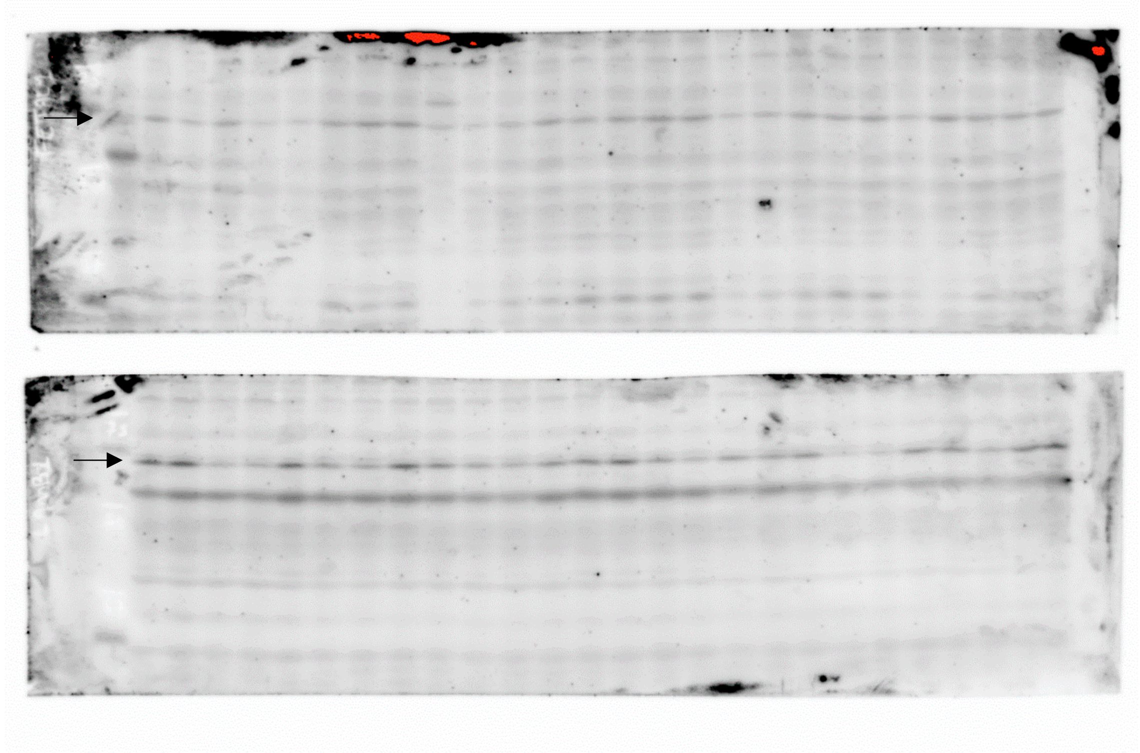

S16: B-actin: Fetal (upper) and Lamb (lower)

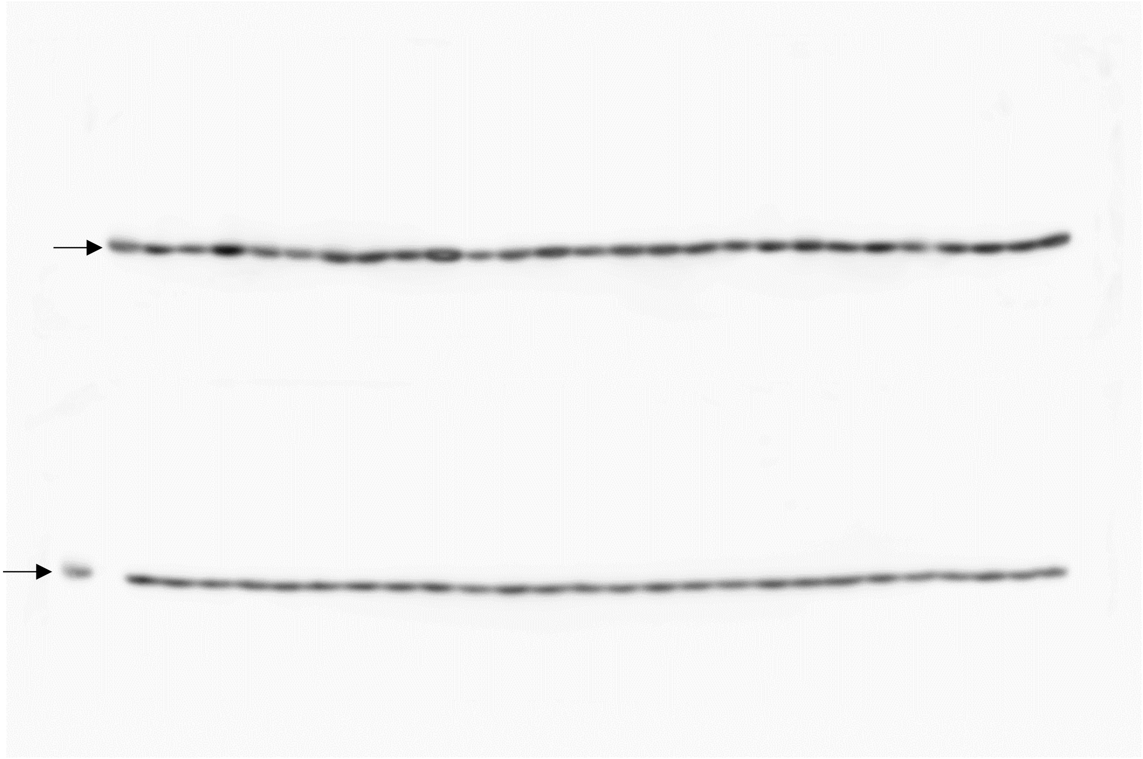

Supplement: Supplementary file 1 [file pharmaceutics-17-00285-s001.zip › supplementary.pdf]
